# Relationship between circulating syndecan-1 levels (CD138s) and serum free light chains in monoclonal gammopathies

**DOI:** 10.1186/s13046-015-0155-4

**Published:** 2015-04-23

**Authors:** Giovanni Cigliana, Eleonora Torti, Francesca Gulli, Elena De Santis, Maria Teresa Dell’Abate, Luigi Colacicco, Francesco Pisani, Laura Conti, Umberto Basile

**Affiliations:** Department of Prevention and Diagnostic Oncology, Laboratory of Clinical Pathology –National Cancer Institute “Regina Elena”, Rome, Italy; Department of Laboratory Medicine, School of Medicine, Catholic University of the Sacred Heart, Largo A. Gemelli 8, Rome, ZIP CODE: 00168 Italy; School of Medicine - Institute of Internal Medicine, Catholic University of the Sacred Heart, Rome, Italy; Hematology and Transplantation, Italian National Cancer Institute “Regina Elena”, Rome, Italy

**Keywords:** Syndecan I, Free light chains, Multiple Myeloma

## Abstract

**Background:**

Monoclonal gammopathies encompass a wide range of diseases characterized by the monoclonal expansion of a B-cell clone. Despite emerging therapeutic strategies, chances of survival of patients who are affected are still scarce, which implies that new tools are necessary not only for the diagnosis but also for the follow-up of patients affected by such diseases. In this context, the use of free light chains (FLCs) has been incorporated into many guidelines.

Likewise, tumor microenvironment is consistently gaining importance as role player in tumor pathogenesis. Specifically, Syndecan-1 (CD138), a heparan-sulfate proteoglycan is attracting interests as it is highly expressed and shed by myeloma plasma-cells.

The aim of our study was to analyze CD138 levels in the serum of patients affected by multiple myeloma or light chain only disease, and to compare the values obtained with free light chain (FLC) kappa, lambda and FLC ratio in both groups of patients.

**Methods:**

84 patients affected by Multiple Myeloma and Light Chain Myeloma were recruited for this study. Serum CD138 was assessed by ELISA (Diaclone Research, France) and FLC values were quantified by nephelometry (Freelite TM Human Kappa and Lambda Free Kits, The Binding Site, UK). Data was analyzed by GraphPad Prism software and Statgraph.

**Results:**

We observed higher CD138 mean values in myeloma patients compared to the light chain only myeloma group. A positive linear regression of CD138 and FLC was observed in the light chain only cohort as opposed to myeloma patients which show an inverse trend.

**Conclusions:**

The study highlighted an existing relationship between FLCs and CD138 and wishes to seek also a correlation in order to rapidly and efficiently perform diagnosis and different diagnostic schemes.

## Introduction

Monoclonal gammopathies encompass a wide range of diseases, ranging from Monoclonal Gammopathies of Uncertain Significance (MGUS) to other M-protein related disorders, thus representing an increasingly growing global issue as they account for an elevated amount of cancers affecting the elderly [1]. They are characterized by the presence of a Monoclonal Component (MC) produced by a B-cell clone in expansion. The dysregulated B cell clone proliferates and secretes either parts or intact immunoglobulins (Igs), identifiable in the patient’s serum as a monoclonal Ig peak by electrophoresis (EF). Most monoclonal peaks imply MGUS, which affects approximately 3.5% of the population over 50 years of age with an average risk of progression to Multiple Myeloma (MM) or, to a lesser extent, to other lymphoproliferative disorders of 1% per year [2-4]. As the elderly population is increasing, novel therapies and strategies have greatly improved patient management. Yet, survival prognosis remains poor, with a 5-year relative survival rate of 35–37% in newly diagnosed MM patients [5-8].

Consequently, these diseases remain incurable. Despite asymptomatic emergence, symptoms often intensify as the disease evolves, substantially reducing health-related quality of life [9-12]. Monoclonal gammopathies are often diagnosed secondarily to other investigations, usually by chance. As population screening is not contemplated, early diagnosis plays a major role in uncovering the underlying disease before it evolves greater malignant features, since MM is considered to be mostly sensitive to treatment at diagnosis. Within this context, diagnostic, monitoring and follow-up tools play a key role in guaranteeing an adequate support.

Recently, FLCs have been proposed and accepted as novel diagnostic and follow-up tools by the global scientific community, and their use has been incorporated in most guidelines both at national and international levels [13-20]. Nevertheless, the assays are still lacking an internationally validated standard, which implies that results are often discrepant [21,22]. Commercially available FLC assays represent a major improvement in monoclonal gammopathy detection and monitoring. However, some critical aspects of both assays still require amelioration, mainly due to the intrinsic characteristics of FLCs and their elevated variability. These, as well as other issues, such as the elevated ability of FLCs to form dimers, tetramers and other conformations, cause underestimation from nonlinear reactions as well as overestimation, possibly due to a lack of parallelism between antigen and antibody, as well as to an unknown antigen excess effect [23-26].

It is therefore increasingly important to seek markers that are indicative of initial malignant proliferation, in order to achieve differential diagnosis and optimal therapeutic indications in the quickest possible manner. In this light, tumor microenvironment (as well as the role of exosomes in creating a cancer-fostering niche) has recently led to new strategies and opened up new therapeutic scenarios [27].

Of particular interest is the interplay between CD138 and its role in tumor biogenesis. CD138 is a cell surface transmembrane heparan sulfate proteoglycan expressed by a variety of cell types. Intact CD138 behaves as cell signaling mediator at different levels involving different parts of the molecule [28]. Cell surface CD138 is processed by a specific heparanase endo-β-D-glucuronidase consequently releasing the extracellular, bioactive portion (ectodomain) of the molecule [29]. Normally, CD138 is constitutively shed at low levels by cells, although the process is accelerated under specific circumstances and stimuli, as well as in malignant contexts [30-32]. Elevated levels of this proteoglycan have been reported in lung cancer, Hodgkin’s Lymphoma as well as in MM [33-35].

Results from in vitro and in vivo studies suggest a role of soluble CD138 in promoting tumor growth as well as tumor cell dissemination. For this reason, the CD138/heparanase axis is consistently gaining attention as therapeutic target due to its importance in driving cancer and in determining tumor aggressiveness [36,37]. Myeloma cells in the bone marrow, as well as circulating plasma cells, specifically express high levels of CD138, thus making them the main source of soluble CD138 in the context of this disease [38,39]. Shed CD138 may remain soluble or it may accumulate in the extracellular matrix [40]. Once shed, CD138 exerts its effects possibly by fostering the tumor microenvironment by inducing activation of signaling molecules [41,42]. A limited yet consistent body of literature reports that serum levels of CD138 in myeloma patients may be considered an independent predictor of poor prognosis for patients [43] and a reliable prognostic factor at different phases of the disease [44,45]. In addition, CD138 shedding is also chemotherapy-induced and a drug-induced relapse of FLCs has also been observed [46-48]. Moreover, the same studies reported a notable extramedullary effect of FLCs following therapy administered to myeloma patients, pointing to an effect of therapies on tumor microenvironment. It is therefore of uttermost importance to consider CD138 interaction with the tumor microenvironment and its effects on the disease prior to drug administration in myeloma patients.

The aim of our study was therefore to analyze CD138 levels in the serum of patients affected by Intact Immunoglobulin Multiple Myeloma or Light Chain Multiple Myeloma and to compare the values obtained with serum FLC kappa (κ) or lambda (λ) chains involved in both groups of myeloma patients. This was done in order to evaluate the clinical utility of serum levels of CD138 in the differential diagnosis of monoclonal gammopathies as a parallel biomarker to be used in association with the FLC assay, especially in the light of therapy administration and monitoring relapsing effects.

## Materials and methods

### Patients

At the time of the present study, 84 patients (40 women, 44 men, mean age = 63,4 yrs, ±11.6 yrs) affected by MM and light chain disease were available for analysis and were recruited in our center at the National Cancer Institute “Regina Elena” of Rome from 2010 to 2013. Inclusion criteria were: presence of monoclonal peak by EF, confirmed by serum immunofixation electrophoresis (sIFE) as well as urine immunofixation (uIF). Exclusion criteria included: presence of renal failure, presence of inflammatory diseases or infections. All subjects were enrolled in the study during clinical check-ups performed in our center.

The nature of the study was explained to both groups enrolled. Blood samples were collected once patients provided their informed consent (in accordance with the Principles of the Declaration of Helsinki, 6th revision of Edinburgh, 2000). An aliquot was retained to be tested for CD138 and all assays were performed in the laboratory premises of the Catholic University “A. Gemelli” of Rome. Patients were then grouped according to the myeloma type and serum samples were obtained and recorded upon performing check-ups, just before therapy. Patients were then stratified into subgroups, according to disease type and according to the type of MC at the time of the first evaluation (either Intact Immunoglobulin Multiple Myeloma with kappa or lambda light chains or only Light Chain Multiple Myeloma with either kappa or lambda light chains, according to the International Myeloma Working Group Guidelines). Components were determined by means of serum protein EF, sIF and uIF. Bone marrow fine needle aspirates were performed and the diagnosis was assessed according to International Myeloma Working Group Guidelines. Patient characteristics are reported in Tables 1 and 2.

**Table 1:** **Characteristics of the two groups of pathological patients**

	**Light Chain MM**	**Intact Immunoglobulin MM**	**Total**	**Mean Age**
Females	16	24	40	63 ± 12
Males	18	26	44	62 ± 10
Total	34	50	84	63 ± 11

Age is reported as Mean ± standard deviation.

**Table 2:** **Distribution of clonality within the Intact Immunoglobulin Multiple Myeloma cohort**

**Intact Immunoglobulin MM Clonality**	**Number of Individuals**
IgG	31
IgA	2
IgM	11
IgD	1
Biclonal	5
Total	50

Control samples were obtained from 40 healthy age-matched and sex-matched individuals (blood donors). All controls were previously tested for the presence of MC by sEF and by both sIFE and uIF (Sebia, France). Inclusion criteria were: absence of monoclonal peak by EF and bands on IFE, as well as normal levels of C-Reactive Protein (CRP). FLC determination on control samples was omitted, as there would be no light chain involved, and FLC ratios would fall within normal ranges.

### Quantification of CD138s and FLCs

#### CD138s quantification assay

All samples were assayed at the same time for CD138 and sFLC. Serum CD138 levels were determined using ELISA kits specific for human CD138 (Diaclone Research, France). The assay was performed according to the manufacturer’s instructions, as follows: 100 ml of serum were added to pre-coated wells and incubated with anti-CD138 biotinylated antibody. The wells were washed and horseradish peroxidase–streptavidin conjugate was added. After washing, the substrate was added, the reaction was stopped and the absorbance was read at 450 nm. Serum concentrations of CD138 were expressed as ng/mL. CD138 levels were measured at least twice for each patient, with reproducible results. The assay was performed on the SKYLAB (DASIT, Italy) automatic system.

#### FLCs evaluation

Serum FLCs were assessed by means of Turbidimetric assay (Freelite TM Human Kappa and Lambda Free Kits, The Binding Site, UK) and performed on the SPAplus instrument (The Binding Site, UK). Samples were tested according to the manufacturer’s instructions and serum dilutions, where necessary, were performed according to the manufacturer’s recommendations.

### Statistical analysis

Collected data sets were reported on Microsoft Excel™ worksheets (Microsoft, Redmond, WA, USA) and analyzed by Prism GraphPad (La Giolla, CA, USA) and Statgraph.

Graphical interpretation of value distribution was achieved by plotting Box-and-Whisker plots (in order to show data distribution of the two pathological cohorts of patients). Global data distribution was shown by plotting scatter graphs of the three groups analyzed. Statistical data analysis was performed using Student’s t-test.

**Figure 1:**
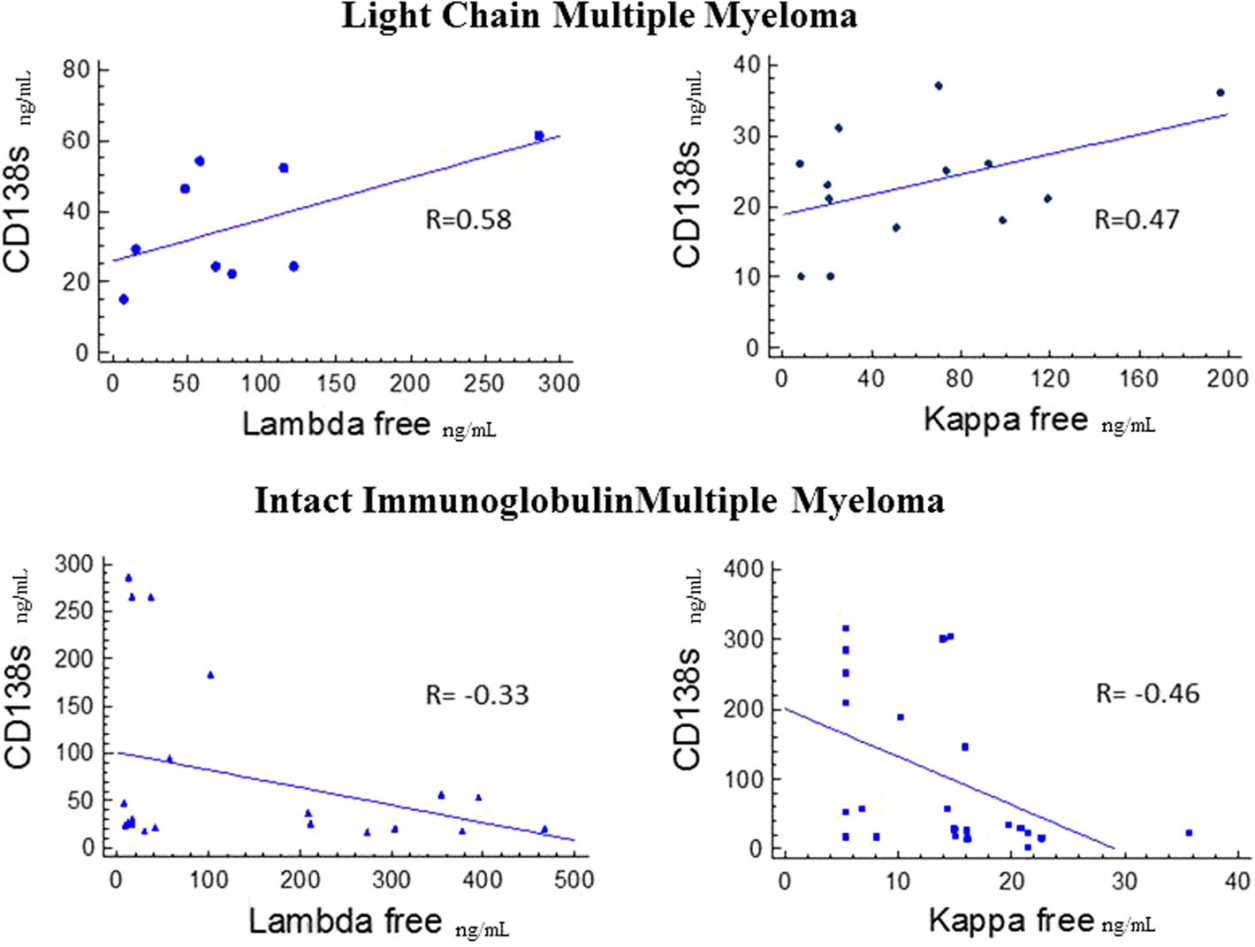
Scatter graph and correlations (R) of serum CD138 concentrations versus Serum Free Light Chain Kappa or Lambda concentrations in Intact Immunoglobulin Multiple Myeloma and Light Chain Myeloma Patients. R values were calculated by Pearson’s correlation.

**Figure 2:**
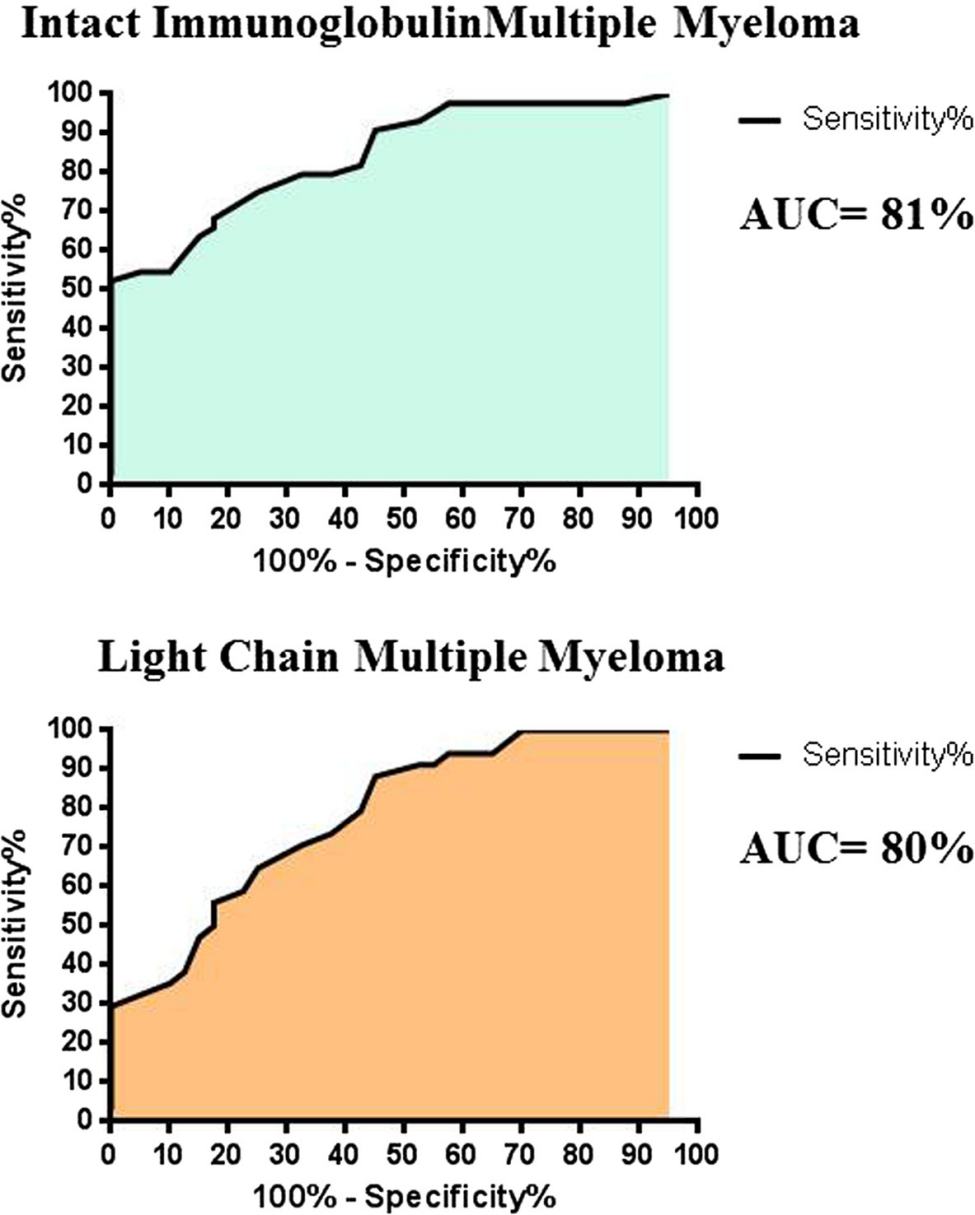
ROC curves depicting percentage Specificity and Sensitivity of serum CD138 concentration values in Intact Immunoglobulin Multiple Myeloma versus serum CD138 concentration values in controls, as well as serum CD138 concentration values in Light Chain Multiple Myeloma versus serum CD138 concentration values in controls.

Analysis of linear correlations was performed by means of Pearson’s correlation, in order to assess the degree of association of the two variables. Correlation coefficients are reported in Figure 1. ROC curves (Receiver Operating Characteristic or Relative Operating Characteristic curves) were generated by plotting data (CD138 values of patients against CD138 values of healthy donors) on GraphPad and the resulting information was then re-plotted on Excel (Figure 2). Data from serum CD138 concentrations in healthy subjects were plotted against values derived from the two groups of patients to give estimates of true positive values (sensitivity) and the proportion of false negatives (specificity). Plotted values are represented as a curve, and the Area Under the Curve (AUC) is indicative of diagnostic accuracy. An AUC = 1 (100%) denotes full accuracy of the test. In this way, it is possible to discriminate normal from abnormal values, which give an estimate of a cut-off value for a specific test in a particular setting. Usually, an AUC = 80% is considered as optimal value. Results were deemed statistically significant for P < 0.05.

## Results

Samples from the first clinical evaluations were considered: serum from routine blood tests at initial diagnosis was used for CD138 and FLC quantifications.

**Figure 3:**
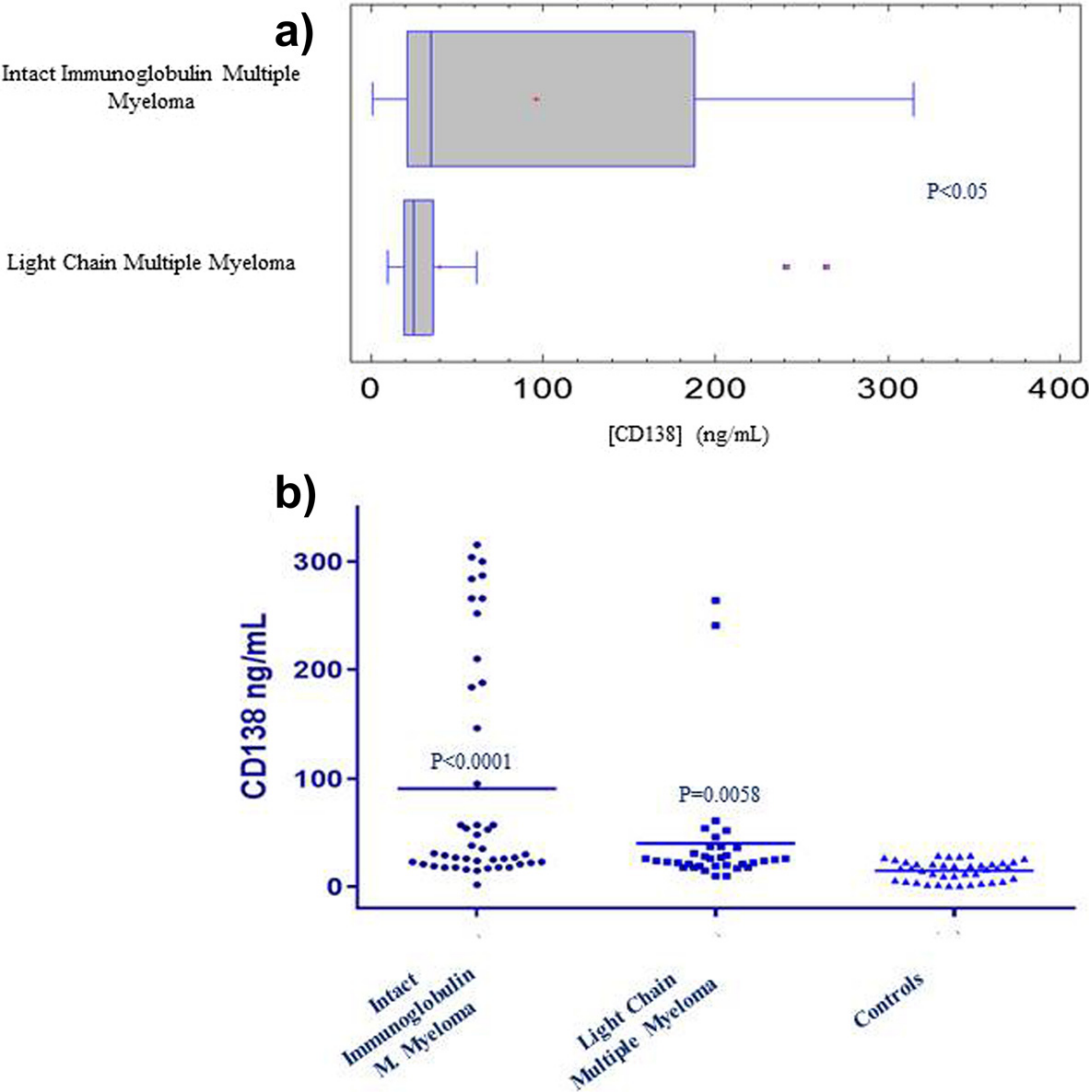
Distributions of CD138 concentrations **a.)** Box and Whisker plot depicting the distribution of CD138 serum concentrations in Intact Immunoglobulin Multiple Myeloma Patients and Light Chain Multiple Myeloma patients. Data in the graph depict the following values: *Intact Immunoglobulin Multiple Myeloma (Median = 30.5). **Light Chain Multiple Myeloma (Mean = 24.5). **b.)** Scatter plot depicting CD138 concentrations among the two groups of patients compared to healthy controls. (Intact Immunoglobulin Multiple Myeloma vs Light Chain Multiple Myeloma P = 0.012; Intact Immunoglobulin Multiple Myeloma vs Controls P < 0.0001; Light Chain Multiple Myeloma vs Controls P = 0.006). Data in the graph depict the following values: *Intact Immunoglobulin Multiple Myeloma (Mean = 90.73, SEM = 15.63, SD = 103.80). **Light Chain Multiple Myeloma (Mean = 40.21, SEM = 9.47, SD = 55.22). ***Controls (Mean = 15, SEM = 1.43, SD = 9.02). (P values were calculated by Student’s Unpaired t-test).

The distribution of serum CD138 concentrations is depicted in Figure 3a. and b. Box-Plot analysis of data reveals two different distributions of sCD138 concentration values among the two pathological groups. Similarly, a statistically significant difference was observed between CD138 concentrations of both groups, as depicted in Figure 3b. As expected, we observed higher serum CD138 concentrations in the Intact Immunoglobulin Multiple Myeloma subset of patients compared to Light Chain Multiple Myeloma patients: mean CD138 values in Intact Immunoglobulin Multiple Myeloma patients was 90.7 ng/mL, whereas 40.2 ng/mL in Light Chain Multiple Myeloma patients (P =0.012). By contrast, control samples mean CD138 concentration was 15.0 ± 9.0 ng/mL (range = 1-29 ng/mL). Increased sCD138 values observed within both cohorts of myeloma patients (as opposed to the control group) are in line with reports from other studies [43-45].

Light Chain Multiple Myeloma patients show positive linear correlation coefficients for sCD138 and involved FLC, as opposed to Intact Immunoglobulin Multiple Myeloma patients, in which sCD138 levels show a decreasing trend when compared to involved FLCs (Figure 1). Not surprisingly, ROC curves performed for each subgroup of patients versus sCD138 quantification in controls were highly significant (P < 0.001). Extrapolated data from ROC analysis plotted on graphs show that specificity and sensitivity plots intercept at exactly 75%, for a corresponding sCD138 value of 21.50 ng/mL. 100% sensitivity is first seen for sCD138 values of 29.50 ng/mL, whereas 100% specificity corresponds to sCD138 values of 1.5 ng/mL. On the other hand, Light Chain Multiple Myeloma specificity and sensitivity plots intercept at sensitivity = 67.5 with a corresponding sCD138 value of 20.5 ng/mL, and at specificity = 70.59%, which corresponds to sCD138 values of 20.50 ng/mL. 100% sensitivity is seen at sCD138 values = 30.00 ng/mL and specificity at sCD138 values = 9.00 ng/mL. Intact Immunoglobulin Multiple Myeloma show 95% specificity for sCD138 values = 15.50 ng/mL, whereas Light Chain Multiple Myeloma 95% specificity is represented by sCD138 values = 10.00 ng/mL.

## Discussion

Our results suggest a possible role of sCD138 as a prognostic marker to be implemented for further use in the context of monoclonal gammopathies, and in particular for what concerns MM. A good prognostic system in MM should ideally form the basis upon which the best treatment can be selected. It should therefore only include variables containing independent prognostic information. In order to be useful within clinical practice, these should be available at diagnosis and be measured with simple reproducible techniques. A number of prognostic factors reflecting various aspects of the disease have been identified in myeloma, relating to either the intrinsic malignancy of the tumor, host-tumor interactions, renal function, or tumor mass. Of these, FLCs concentration is regarded as one of the most powerful prognostic factors. In our study, we show that sCD138 provides substantial prognostic value in a correlation model with FLCs, considered as valuable prognostic markers. Our findings uncover an existing relationship between the FLCs involved and CD138 shedding, and the different trends in relation to each other.

As depicted in Figure 1, two different correlations characterize each group of patients when considering sCD138 against the involved FLCs. Light Chain Multiple Myeloma shows increasing amounts of sCD138 proportionally to a corresponding increase of serum FLCs. On the contrary, serum of Intact Immunoglobulin Multiple Myeloma patients shows correspondently lower levels of CD138 compared to involved FLCs concentration increase. A stronger trend was generally observed for correlations of FLCs and sCD138 in Light Chain Multiple Myeloma (R _к/CD138_ = 0.47and R _λ/CD138_ = 0.58) whereas this was not so evident in the Intact Immunoglobulin Light Chain Myeloma cohort (R _к/CD138_ = 0.46 and R _λ/CD138_ = 0.33). One possible explanation could be due to the elevated variability of the FLC test, due to the intrinsic properties of the light chains themselves. This is particularly true for lambda chains, which are more susceptible to assay variability due to their properties and structure. In fact, lambda chains are incline to polymerization and all commercially available assays show variability in their detection. Thus, the dispersion of values may reflect the intrinsic characteristics of the FLC.

**Figure 4:**
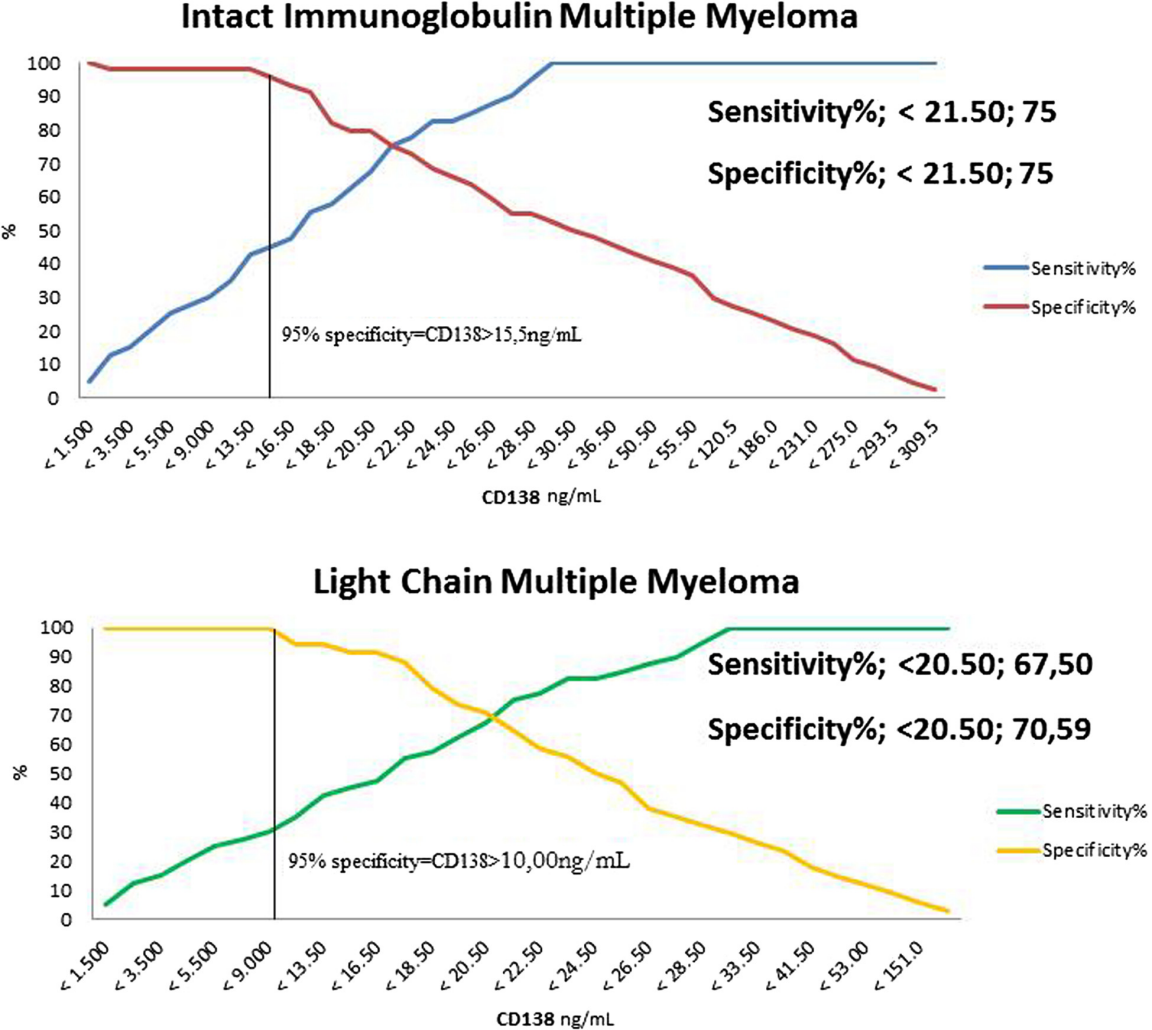
Data from ROC curve analysis vs CD138 concentrations in Intact Immunoglobulin Multiple Myeloma or Light Chain Multiple Myeloma. CD138 concentrations corresponding to 95% discrimination specificity of the test are reported within each graph. The black line indicates the CD138 value corresponding to 95% specificity.

Specificity and sensitivity of sCD138 was tested in order to determine test accuracy, as well as to have an estimate of the cut-off values for this test. Specificity and sensitivity of sCD138 in both types of myeloma were evaluated in order to assess normal and abnormal values. Specificity at 95% show that sCD138 measurement is capable of detecting Intact Immunoglobulin Multiple Myeloma for values of sCD138 > 15.5 ng/mL (Figure 4, Intact Immunoglobulin Multiple Myeloma, red line plot), whereas Light Chain Multiple Myeloma are readily detected with a 95% specificity for values of sCD138 > 10.00 ng/mL (Figure 4, Light Chain Multiple Myeloma, yellow line plot). These results are possibly indicative of a differential role played by CD138 shedding and FLCs release in the two pathologies, which could be exploited as diagnostic marker in order to achieve a faster and more efficient result. As CD138 shedding has already been signaled as good prognostic factor in this context [43-45], it is plausible that further studies may be able to elaborate algorithms by merging FLC analysis and shed CD138 which may significantly improve diagnosis, as well as follow-up and patient monitoring.

As observed in our study, CD138 shedding varies differently according to FLCs released by myeloma cells. Similarly, myeloma B-cell clones produce a conspicuous amount of immunoglobulins, which is accompanied by an altered secretion of FLC as well as by an equivalent variation of the normal κ/λ ratio, that is at the core of the FLC quantification assay. Serum FLCs assays rely on the assumptions that serum concentrations of an unbalanced production of FLC in a monoclonal gammopathy setting yields an altered FLC κ/λ ratio. This is considered to be a diagnostic aid, especially in those cases in which the MC is not of great entity. Nevertheless, immunoglobulin light chains must exit cells in order to circulate freely: in this context, it is of great interest to consider the role of CD138 involved in exosome biogenesis, as these cargo containing vesicles seem to promote cancer development as well as other pathological conditions [49]. In light of these findings, it may be appealing to speculate that the expression and shedding of CD138 as well as FLC release into serum may be intertwined. Furthermore, their quantification in myeloma patients may not only be of diagnostic aid, but may also guide towards a more appropriate therapeutic approach while considering the physio-pathological effects of both molecules [50,51].

Indeed, MM is characterized by a profoundly altered equilibrium of factors involved in cell-cell and cell-matrix interactions, which fosters tumor growth and lytic bone lesions [52]. Similarly, exosome biogenesis is significantly up-regulated and exosome protein composition is also particularly altered within this context [51]. Moreover, as CD138 seems to be involved in membrane dynamics and exosome biogenesis, it is of particular interest to explore the matter in view a significant increase in microvesicle production observed in MM and Amyloidosis affected patients. In addition, the same study reported are routing process of FLCs via microvesicles and exosomes [53]. In fact, these structures are often laden with cargo (miRNA, mRNA, lipids, proteins) possibly represented by tumor-specific molecules [54]. Several studies have pointed to the importance of the effects of commonly used chemotherapy drugs on CD138 shedding [54] as well as a notable rebound effect of the drugs on FLC production in these diseases [46; 47]. Therefore, the simultaneous quantification of these two markers may prove a precious diagnostic aid and suggest the best therapeutic choice in a patient-centric manner. Furthermore, as sCD138 itself is not as variable as FLCs, the quantification of this molecule could represent a valuable tool to be used alongside with FLCs quantification during follow-up and monitoring of myeloma affected subjects undergoing treatments. This could be done in order to investigate how different types of myeloma may respond to therapies by shedding a potential tumor-fostering molecule, thereby limiting beneficial effects of the drugs. Besides, since both commercially available assays for FLCs quantification are still lacking standardization and have yet to solve critical issues due to the enormous intrinsic variability of FLC molecules themselves (which make them a very unstable and cumbersome analyte to quantify) it is appealing to consider implementing FLCs quantification with this marker as test adjuvant during diagnosis, follow-up and monitoring of monoclonal gammopathies. Cleaved, sCD138 is in fact less prone to individual variability as opposed to FLCs, and this may greatly simplify the detection of this molecule, thus rendering it a more stable marker to be used alongside standard testing. As it is of vital importance not to miss out any diagnosis [55], these two tests could, in the near future, be used as complementary tools in assisting patient management, particularly in the context of monoclonal gammopathies.

## Conclusion

Our study, albeit preliminary, seeks a correlation between FLCs and soluble CD138. This would enable to perform diagnosis rapidly and efficiently, especially in those cases in which the MC is hardly detectable, but also in consideration of performing different diagnostic schemes. As it was shown that commonly used chemotherapy drugs cause CD138 shedding and that relapse may often be aggressive and lead to death, it is important to consider the effects of this molecule. Shed CD138 offers the advantage of a greater inter-individual antigenic stability, as opposed to FLCs which are highly variable and susceptible to major modifications. It is therefore of absolute importance, in light of these results, to continue the study of sCD138 in the context of MGUS patients and to follow fluctuations of both FLCs and sCD138 in time, so as to detect the switching point to malignant evolution. Further studies are needed in order to clarify the clinical utility of these molecules as markers, and how they may be used not only for an initial diagnosis but also for differential diagnosis. Moreover, future perspectives are aimed at evaluating a possible role for the two analyzed molecules in precocious disease discrimination before progression. The different trends observed between the two molecules may, in the near future, lead to important discriminatory information regarding what type of MGUS is affecting the patient but also concerning the evolution of the disease. Likewise, studies concerning effects of therapy on tumor microenvironment and on the CD138/heparanase axis are greatly required in this context, in order to assess beneficial effects of administered drugs.

To conclude, our study highlights the importance of these molecules and the necessity of pursuing further studies in order to define the exact role and interplay between sCD138, exosome cargo and the effects on FLCs in myeloma, which may reveal to be a key diagnostic marker.
